# Heterogeneous network propagation for herb target identification

**DOI:** 10.1186/s12911-018-0592-z

**Published:** 2018-03-22

**Authors:** Kuo Yang, Guangming Liu, Ning Wang, Runshun Zhang, Jian Yu, Jianxin Chen, Xuezhong Zhou

**Affiliations:** 1 School of Computer and Information Technology and Beijing Key Lab of Traffic Data Analysis and Mining, Beijing Jiaotong University, Beijing, 100044 China; 2 Guanganmen Hospital, China Academy of Chinese Medical Sciences, Beijing, 100053 China; 3 Beijing University of Chinese Medicine, Beijing, 100029 China; 4 Data Center of Traditional Chinese Medicine, China Academy of Chinese Medical Sciences, Beijing, 100700 China

**Keywords:** Heterogeneous herb-target network, Random walk, Herb target identification

## Abstract

**Background:**

Identifying targets of herbs is a primary step for investigating pharmacological mechanisms of herbal drugs in Traditional Chinese medicine (TCM). Experimental targets identification of herbs is a difficult and time-consuming work. Computational method for identifying herb targets is an efficient approach. However, how to make full use of heterogeneous network data about herbs and targets to improve the performance of herb targets prediction is still a dilemma.

**Methods:**

In our study, a random walk algorithm on the heterogeneous herb-target network (named heNetRW) has been proposed to identify protein targets of herbs. By building a heterogeneous herb-target network involving herbs, targets and their interactions and simulating random walk algorithm on the network, the candidate targets of the given herb can be predicted.

**Results:**

The experimental results on large-scale dataset showed that heNetRW had higher performance of targets prediction than PRINCE (improved F1-score by 0.08 and Hit@1 by 21.34% in one validation setting, and improved F1-score by 0.54 and Hit@1 by 69.08% in the other validation setting). Furthermore, we evaluated novel candidate targets of two herbs (rhizoma coptidis and turmeric), which showed our approach could generate potential targets that are valuable for further experimental investigations.

**Conclusions:**

Compared with PRINCE algorithm, heNetRW algorithm can fuse more known information (such as, known herb-target associations and pathway-based similarities of protein pairs) to improve prediction performance. Experimental results also indicated heNetRW had higher performance than PRINCE. The prediction results not only can be used to guide the selection of candidate targets of herbs, but also help to reveal the molecule mechanisms of herbal drugs.

**Electronic supplementary material:**

The online version of this article (10.1186/s12911-018-0592-z) contains supplementary material, which is available to authorized users.

## Background

Target identification of herb medicine is the primary step toward investigating herbal molecular mechanism and improving clinical efficacy of treatment. Unlike allopathic Western Medicine, Traditional Chinese medicine (TCM) is characterized as holistic emphasizing on regulating the integrity of the human body [1]. The diverse herbal ingredients and multi-target molecular mechanism are critical characteristics of herb medicine [2, 3]. In recent years, herbal molecular mechanism studies mainly are focused on animal experiments, for example, Yu et al. [4] found that SP1 is potential target of turmeric by mice experiments. However, animal experiments not only cost a lot of times and manpower, but also are limited to the scale of minority herbs, which has caused enormous challenge for TCM researchers.

Network pharmacology emphasized the paradigm shift from “one target, one drug” to “network target, multicomponent therapeutics,” highlighting a holistic thinking also shared by TCM [2, 5]. By integrating computational and experimental methods, network pharmacology provided a new perspective of herb compatibility [6] and herbal molecular mechanism research [7]. On the one hand, plenty of medical associations data, i.e., drug-target associations (DrugBank [8] and Stitch [9]), phenotype-genotype associations (OMIM [10] and DO [11]), protein-protein interactions (String10 [12]) and human symptom-disease associations [13], provided abundant and valuable medical data, which can be applied to computational methods to predict herbal protein targets. On the other hand, plenty of prediction approaches based on network propagation have been widely applied to identify genetic associations, e.g., drugCIPHER-MS [14] for drug target identification, PRINCE [15], pgWalk [16] and Know-GENE [17] for disease gene identification and herb target prediction, which indicated that network propagation is an effective approach to figure out the problem of link prediction in complex network. In our previous work [3], we proposed a network-based herb target prediction algorithm integrating efficacy-based herb similarity, which implied efficacy-based herb similarity was better measure of herb correlation than herbal chemical-based similarity.

In TCM field, several curated databases involving the associations between herbal ingredients and targets, i.e. HIT [18], TCMID [19], have been established. Yet, the high credible dataset of herb-target associations is still incomplete. Therefore, developing effective approaches to identifying herbal protein targets has become a key step to decode molecular mechanism of herbs. Network pharmacology methods have been utilized to active ingredient-target networks of herbs that were responsible for the beneficial effects against hepatocellular carcinoma [20]. By integrating serum pharmacochemistry-based screening with high-resolution metabolomics analysis, Wang et al. [21] developed chinmedomics to identify the bioactive constituents of herbs and predicted action potential targets. Zhang et al. [22] proposed a computational strategy for network understanding of herb pharmacology via rapid identification of putative ingredient-target interactions in human structural proteome level. Liang et al. [23] proposed a holistic analysis method combines chemical and therapeutic properties with network pharmacology to decipher targets and ingredients of herbal formulae. Zhao et al. [24] introduced a system pharmacology model based on absorption filtering, network targeting and systems analyses to clarify the active compounds and therapeutic mechanisms of Bufei Jianpi formula.

**Fig. 1:**
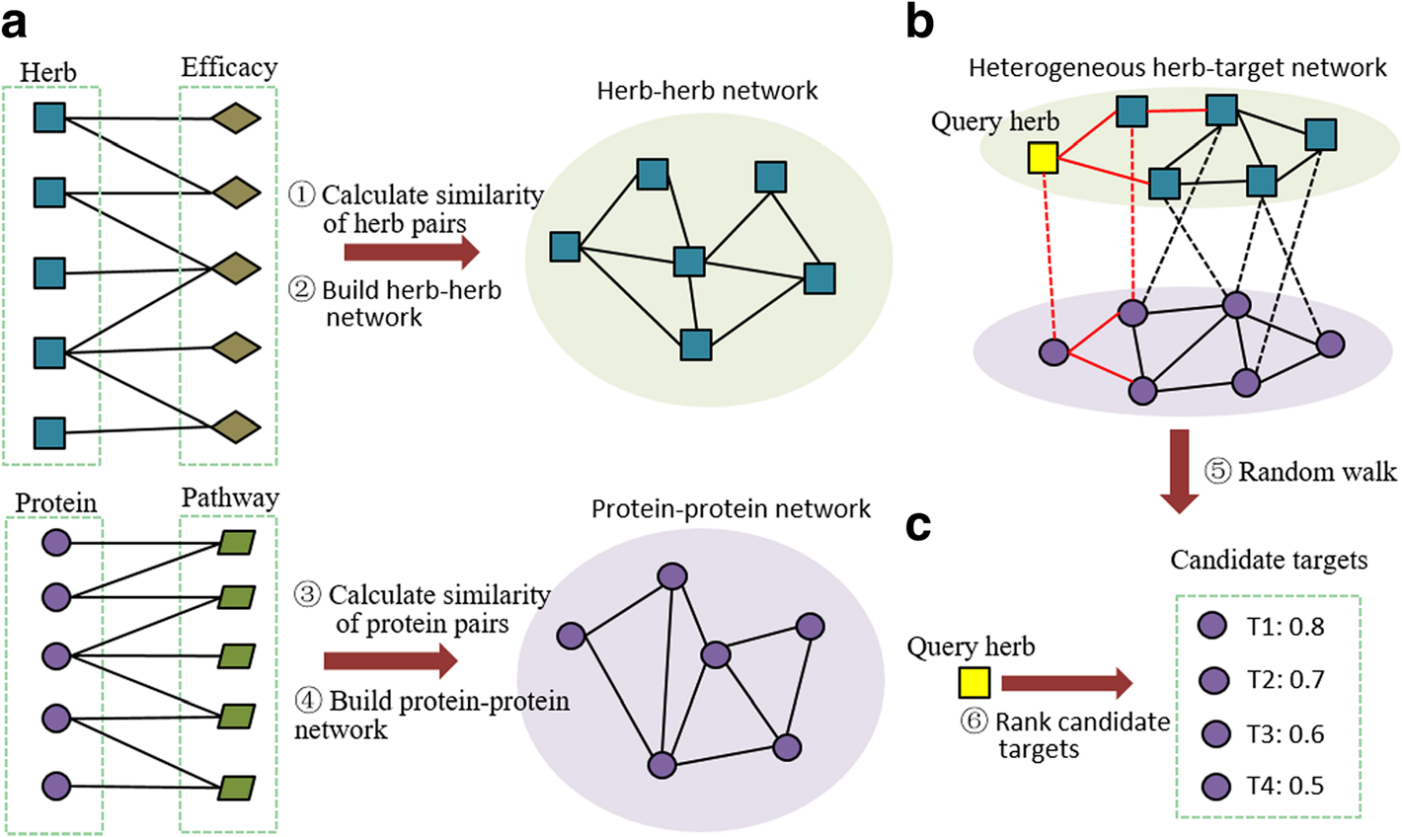
The workflow of heNetRW. Based on herb-efficacy and protein-pathway associations, cosine similarities of herb pairs and protein pairs were calculated. Then the herb-herb network and protein-protein network (Fig. 1**a**), whose nodes represent herbs or proteins and edges represent shared efficacies or shared pathways, were built. Given a query herb, the random walk algorithm was simulated (red line) on the heterogeneous herb-target network (Fig. 1**b**). By ranking the candidate targets by correlation scores, candidate targets of a given herb can be identified (Fig. 1**c**)

Here, aiming at protein targets identification of herbs, a random walk algorithm [25] on heterogeneous herb-target network (heNetRW) was put forward (Fig. 1). By constructing a heterogeneous herb-target network and simulating random walk on the network, the candidate targets of the query herb can be predicted. In the experimental stage, two validation setting: NoTarget and HalfTarget situations were simulated to evaluate prediction performance of heNetRW. And the final results indicated that heNetRW has better performance than baseline algorithm PRINCE (improved F1-score by 0.08 and Hit@1 by 21.34% under NoTarget simulation, improved F1-score by 0.54 and Hit@1 by 69.08% under HalfTarget simulation). Furthermore, we evaluated novel candidate targets (not recorded in the benchmark) of rhizoma coptidis and turmeric by consulting recent published papers and conducting shortest path analysis, whose results manifested the correlativity between these candidate targets and query herbs.

## Methods

### Dataset

**Fig. 2:**
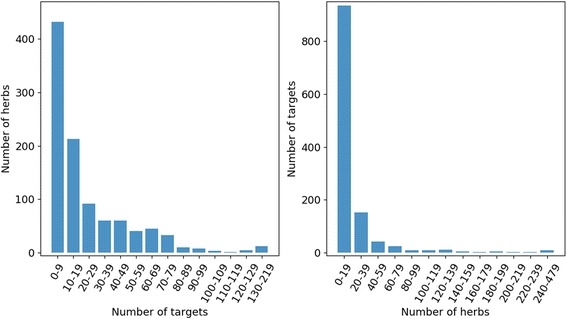
The distribution of associations between herbs and targets. The target number distribution of herbs were shown in Fig. 2**a**. The horizontal axis represents number of targets, and the vertical axis represents the number of herbs with corresponding number of targets. Fig 2**b** showed the targeted herb number distribution of targets. The horizontal axis represents number of herbs, and the vertical axis represents the number of targets with corresponding number of targeted herbs

We integrated 1427 herbs were extracted from HIT [18] and Chinese pharmacopoeia (CHPA, 2015 edition) and 16,005 proteins from HIT and KEGG [26] databases. In the HIT database, there are associations between herbal ingredients and targets from medical literatures. By integrating herb-ingredient and ingredient-target associations, 23,453 associations between 1016 herbs and 1214 targets (see Additional file 1) were connected directly through 511 herbal ingredients. On average, each herb was associated with 23.08 targets, and each target was relevant to 19.32 targeted herbs (Fig. 2). The target number of 57.48% herbs was bigger than ten, which also verified multi-target mechanism of herbs. Otherwise, we collected 3487 herb-efficacy associations between 742 herbs and 360 efficacies from CHPA (see Additional file 2). 16,162 protein-pathway associations between 4794 proteins and 244 pathways (see Additional file 3) were also collected from KEGG database.

### Similarity calculation of herb pairs and protein pairs

By building efficacy or target vectors and measuring the cosine value of these vectors, efficacy-based and target-based similarities of herb pairs can be calculated. For example, given *m* herbs and *n* related efficacies, every herb *i* can be represented by a vector of efficacy *V*_*i*_ = (*w*_*i*, 1_, …, *w*_*i*, *j*_, …, *w*_*i*, *n*_), where *w*_*i*, *j*_ = 1 if efficacy *j* belong to herb *i*, if not, *w*_*i*, *j*_ = 0. Then the efficacy-based similarity of herb *x* and *y* can be measured by the cosine value of their vectors (Eq. 1). Similarly, target-based herb similarities were also calculated.

1 $$ Cos\left({V}_x,{V}_y\right)=\frac{V_x\bullet {V}_y}{\mid {V}_x\mid \bullet \mid {V}_y\mid } $$

Similarly, by building vectors of pathways and herbs, and measuring the cosine value of these vectors, pathway-based and herb-based similarities of protein pairs also can be calculated.

### Herb pairs with similar efficacies indicate similar targets

The basic assumption of heNetRW is that herb pairs with shared efficacies indicated shared targets, and protein pairs with shared pathways implied shared targeted herbs. Given an herb pair, we quantified the efficacy-based cosine similarity between the herb pair, and measured the target-based cosine similarity of the pair as the average pairwise similarity score. We partitioned the similarities of all herb pairs into 10 bins of equal size. The average target-based similarity of herb pairs in each bin can be calculated. Similarly, pathway-based and herb-based similarity of protein pairs were also measured.

To illustrate overlap results of herb pairs and protein pairs, we compared the overlap results with random shuffle [27]. We took herb pairs as an example. First of all, for herb pairs, remaining constant known efficacies of herbs, we shuffled randomly equal number of targets for each herb. Secondly, based on the random targets of herb pairs, the overlap results can be calculated. Finally, the above procedures were repeated for 100 times, and the average ratios of overlap results can be obtained.

### Construction of herb-herb network and protein-protein network

Based on efficacy-based similarities of herb pairs, we constructed the herb-herb network, where nodes represent herbs and edges represent herb pairs with shared efficacies (that is, efficacy-based herb similarities are bigger than zero). The network we built may contain a large number of low confident edges between herbs pairs with small similarities. Therefore, only *α* neighbor herbs with the highest similarity scores for each herb were selected to build a more confident herb-herb network.

Similarly, by calculating pathway-based similarities of proteins, the protein-protein network also can be constructed. For a more confident protein-protein network, *β* neighbor proteins with the highest similarity scores for each protein were kept. The parameters *α* and *β* have been tuned to observe the influence of target prediction under different values of *α* and *β*.

### Random walk on heterogeneous herb-target network to identify candidate targets

Given an herb-herb network, a protein-protein network and known herb-target associations, the heterogeneous herb-target network (HHGN) can be constructed, which included two type nodes: herb and protein nodes, and three type edges: herb-herb, protein-protein, and herb-target edges. Then the process that a random walker wandered on HHGN can be simulated to identify candidate targets of given herb.

For the HHGN, there are an herb layer, a protein layer, and interconnections between the two layers. The herb layer, which can be weighted by efficacy-based similarities of herbs, was composed of herbs and their relationships. The protein layer, which can be weighted by pathway-based protein similarity, was composed of proteins and their associations. Interconnections, which connected herb layer and protein layer, are composed of known associations between herbs and proteins. Hence, given a query herb, the random walker would start a journey on the HHGN with initial probability ***p***^(0)^. In next each step, the walker would select to start a new journey with the probability *θ* or select to move to neighbors of the current node with the probability 1 − *θ*. For moving to neighbors, the walker would select to jump from the herb layer to the protein layer or vice versa with probability *φ* or select to wander in either the herb layer or the protein layer with the probability 1 − *φ*. After a number of steps, the probability of each node on the HHGN would reach a steady state ***p***^(*t*)^, which can be used to measure the strength between the query herb and candidate targets.

Mathematically, HHGN is denoted by ***I*** = (***H***, ***G***, ***R***), where ***H*** = (*h*_*ij*_)_*m* × *m*_ is the weight matrix of herb-herb network, ***G*** = (*g*_*ij*_)_*n* × *n*_ is the weight matrix of protein-protein network, ***R*** = (*a*_*ij*_)_*m* × *n*_ is the adjacency matrix of herb-protein network, and *m* and *n* is the numbers of herbs and proteins, respectively. The heNetRW is described as follows:

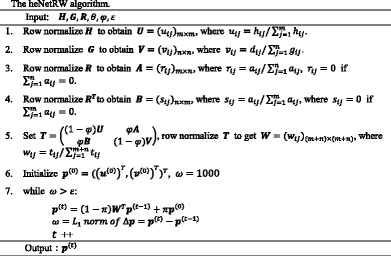



For the transition matrices ***A*** and ***B***, *r*_*ij*_ and *s*_*ij*_ are the probability that the walker jumps from the *i*-th herb to the *j*-th protein and jumps from the *i*-th protein to the *j*-th herb, respectively. ***u***^(0)^ = (*u*^(0)^)_*m* × 1_ and ***v***^(0)^ = (*v*^(0)^)_*n* × 1_ are initial probabilities for the herb layer and the protein layer, respectively. ***p***^(0)^ represents the initial probability of every herb and protein. ***p***^(t)^ represents probability of nodes after *t*-th iteration of random walker, and *∆****p*** represents changed matrix of *t*-th and (*t* − 1)-th iteration.

Finally, when the *L*_1_ norm of matrix *∆****p*** is smaller than *ε*, the algorithm obtained steady-state probability ***p***^(*t*)^, and it contains the two part: herb scores ***u***^(*t*)^ and protein scores ***v***^(*t*)^, which can be regarded as predicted targets of the query herb. Sorting all the predicted proteins by the scores, the top n proteins of ranked list were selected as candidate proteins of the query herb.

### Experimental setting and evaluation

We collected 1016 herbs with known targets, 120 of which only targeted one protein target and the remaining 896 herbs targeted more than one protein target. We filtered overlapped 261 herbs with known targets and known efficacies. In real world, there usually are two situations of target identification: (1) the query herb has no target; (2) the query herb has some targets. Therefore, we simulated the two situations: (1) predict all targets for the query herb whose all targets are removed (NoTarget); (2) predict left targets for the query herb whose half number of targets are randomly removed (HalfTarget). Particularly, if the query herb only has one target, it only can be used to the first situation. If all known targets of the given herb were removed, and the given herb didn’t have any herb neighbors in the heterogeneous herb-target network, the initial value ***p***^(0)^ of the given herb will be zero, which would lead that the random walk algorithm would not spread nodes information to other nodes of the network. So when simulating NoTarget, 261 herbs with herbs and targets neighbors were selected as test herbs. And when simulating HalfTarget, we select 896 herbs with more than one known targets as test herbs. For the two situations: NoTarget and HalfTarget, heNetRW and PRINCE were evaluated by leave one out cross validation (LOOCV): remove all of known targets or half of known targets for the query herb and retain all of known targets for other herbs.

If the query herb had k known targets, the top k proteins of ranked proteins list will be selected as candidate targets of the query herb. For example, given the query herb rhizoma coptidis with 64 known targets, our algorithm would select the top 64 targets of ranked list as candidate targets. On the one hand, precision, recall and F1-score of every query herb were calculated by Eqs. 2 and 3, in which TP, FP and FN represent the numbers of true positives, false positives and true negatives, respectively. On the other hand, the top one hit (Hit@1) rate was also used to evaluate the algorithms. Hit@1 considers that the proportion of the query herbs, whose top one protein of ranked list is the known target of the query herb.

2 $$ Precision= TP/\left( TP+ FP\right); Recall= TP/\left( TP+ FN\right) $$

3 $$ F1=\frac{2\bullet Precision\bullet Recall}{Precision+ Recall} $$

### Shortest path analysis

To investigate the associations between candidate targets and known targets of given herbs, average shortest path length (ASPL) and shortest path length (SPL) distribution between them were conducted. First of all, the shortest path length *SPL*(*t*, *s*) between candidate target *t* and known target *s* (*s* ∈ the known targets set *S*) in the protein-protein interaction (PPI) network can be calculated. Then the ASPL can be calculated by the equation:

4 $$ ASPL\left(t,S\right)=\frac{1}{\left|S\right|}\sum \limits_{s\in S} SPL\left(t,s\right) $$

where |*S*| represented the number of known targets. Otherwise, the distribution of all the SPLs was also calculated and compared with random shuffle. For the random experiment, candidate targets were selected randomly from the PPI network, and the SPL distribution between the candidate target and known targets was calculated. The above process was repeated for ten thousand times, finally, the average distribution of random candidate target and known targets can be obtained.

## Results

### Overview of heNetRW

The core idea of heNetRW is simulating random walk on a heterogeneous herb-target network to identify candidate targets of herbs. As illustrated in Fig. 1, firstly, based on herb-efficacy and protein-pathway associations, cosine similarities of herb pairs and protein pairs can be calculated. After that, the herb-herb network and protein-protein network were constructed. The weight of edges in the two networks depend on similarities of herb pairs or protein pairs. There are 741 nodes and 60,753 edges in herb-herb network (Table 1), whose average degree (the number of neighbors) is 163.98. The protein-protein network with 4794 nodes and 656,681 edges has a higher average node degree (= 273.95). Compared with herb-herb network (network density: 0.22), protein-protein network has the lower network density (=0.06). By integrating herb-herb network, protein-protein network and herb-target associations, the heterogeneous herb-target network (network density: 0.03), which is a very sparse network, can be constructed. Given a query herb, random walk algorithm on the heterogeneous network can be simulated to predict candidate targets. Sorting all predicted targets by correlation scores, the ranking list of candidate proteins can be obtained. LOOCV was used to evaluate the prediction performance. In addition, the PRINCE [3] algorithm, which is a state-of-the-art algorithm for herb target prediction, was adopted as the baseline algorithm in our experiments. With efficacy-based similarities of herb pairs, the PRINCE simulated network propagation on the PPI network to predict herb targets.

**Table 1:** The description of related networks

Network	Number of nodes	Number of edges	Average degree	Network density
Herb-herb network	741	60,753	163.98	0.22
Protein-protein network	4794	656,681	273.95	0.06
Heterogeneous herb-target network	6716	740,887	220.63	0.03

### Herb pairs with similar efficacies indicate similar targets

**Fig. 3:**
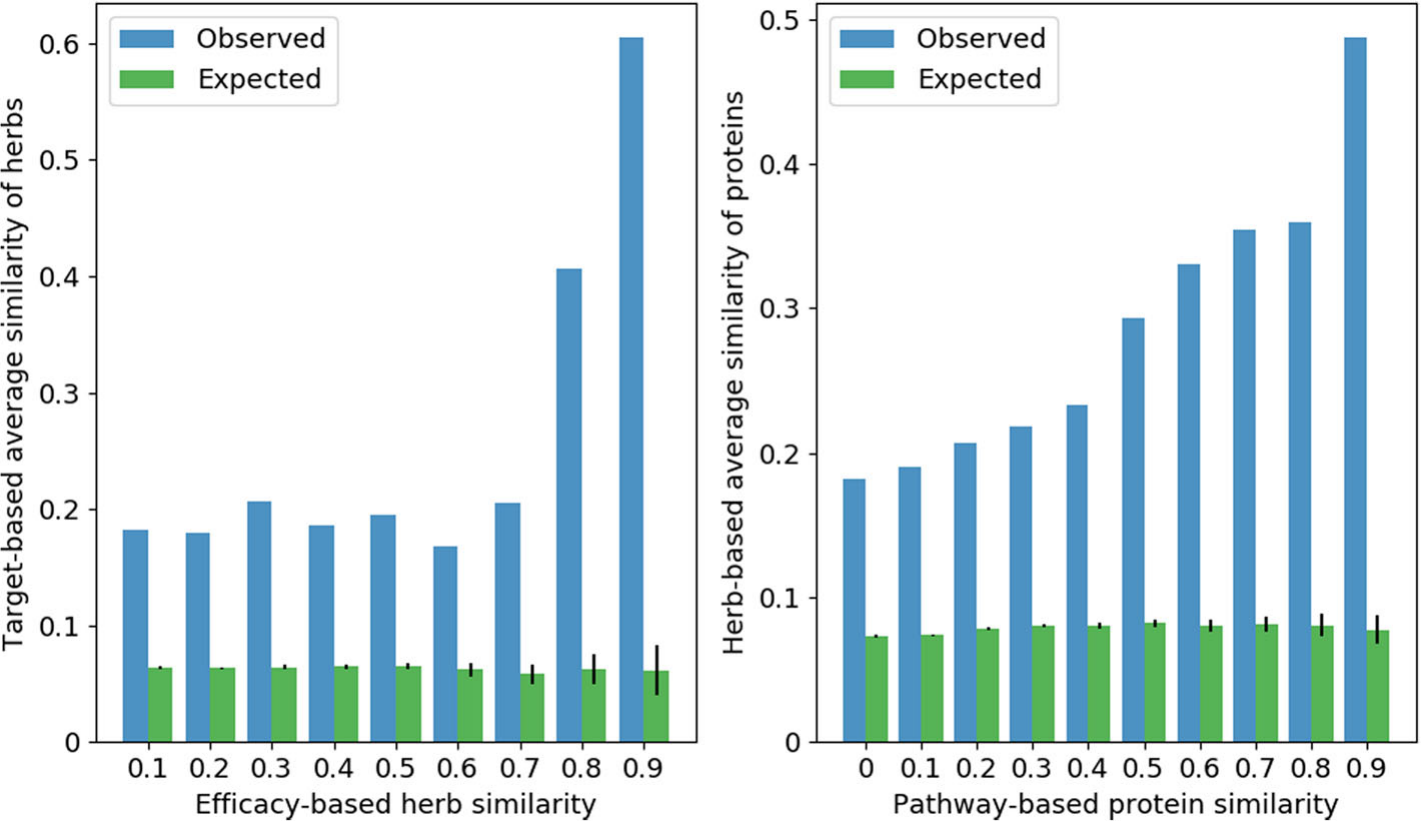
Overlap analysis of herb pairs and protein pairs. Figure 3**a** Herb pairs with shared efficacies imply shared proteins. The horizontal axis represents efficacy-based similarity bins of herb pairs, and the vertical axis represents target-based average similarity of herb pairs under the corresponding efficacy-based herb similarity bins. The mazarine and green bar represent observed results and expected results of random permutation. Figure 3**b** Protein pairs with shared pathways imply shared targeted herbs. Similarly, the horizontal and vertical axis represent pathway-based similarity of protein pairs and herb-based average similarity of protein pairs under the corresponding bins

The basic assumption of our method is that the herb pairs with shared efficacies implied shared targets, and target pairs with shared pathways implied shared targeted herbs. To validate this assumption, we quantified efficacy-based and target-based similarities of herb pairs. And the overlap results were compared with results of random permutation using Fisher-Yates method [27]. Meanwhile, to validate whether protein pairs with similar pathways indicated similar targeted herbs, pathway-based and herb-based similarities of protein pairs also were adopted according to the same way. We selected 261 comon herbs with efficacies and targets as experimental herbs (Fig. 3a). The herb pairs with weak efficacy-based similarities (0.1–0.2) had weak target-based average similarity (0.18; Expected: 0.06±0.0013). Nevertheless, for the herb pairs with strong similarities (0.9–0.1), their target-based average similarity (0.60; Expected: 0.06±0.02) was also strong, which indicated herbs with more similar efficacies are more likely to have more similar targets. Similarly, for 789 proteins with pathways and targeted herbs (Fig. 3b), there were the similar results, which were proteins similarity bin from 0 to 0.1 (0.18; Expected: 0.07±0.0007) versus similarity bin from 0.9 to 1 (0.49; Expected: 0.08±0.01).

### Target identification of herbs

**Fig. 4:**
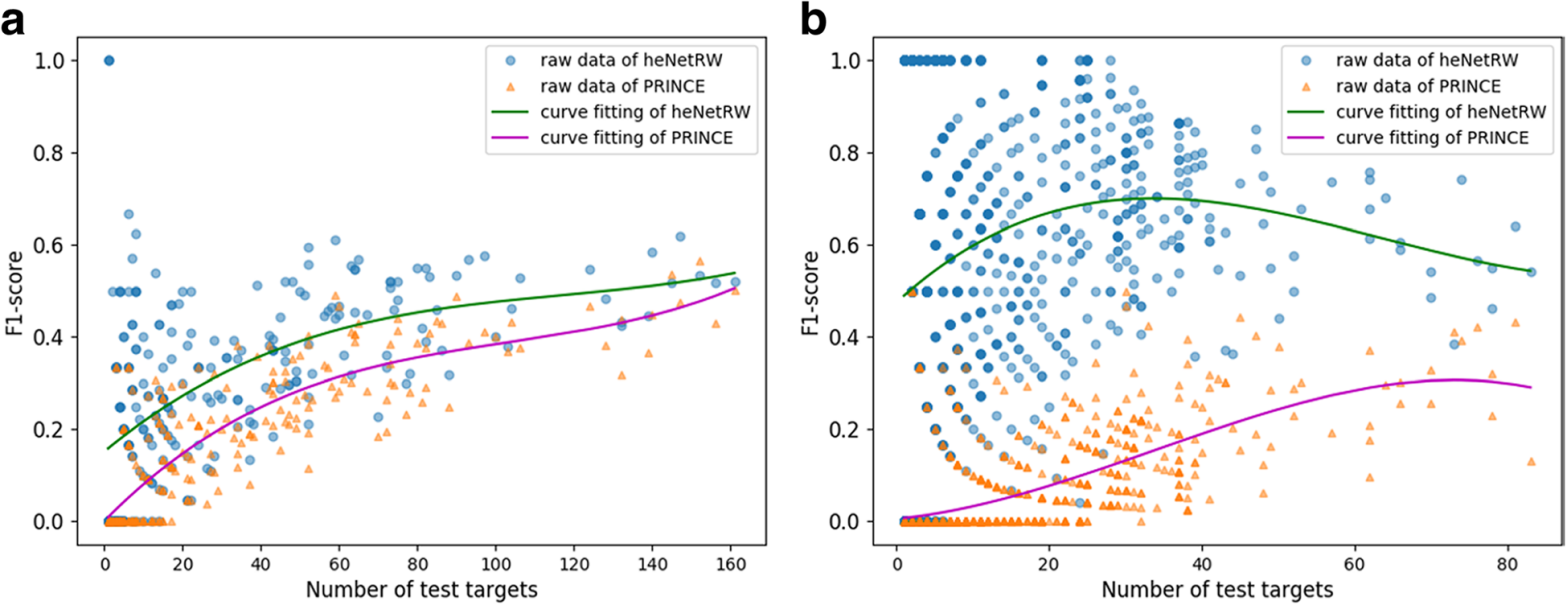
F1-scores distribution of PRINCE and heNetRW. F1-scores distributions of heNetRW and PRINCE under the NoTarget and HalfTarget situations were showed, respectively (Fig. 4**a** and Fig. 4**b**). The horizontal axis represents number of test targets, and the vertical axis represents F1-scores of herbs with corresponding number of test targets. The circle point and triangle point represent the F1-scores of heNetRW and PRINCE. The green and pink line represent the curve fitting of heNetRW and PRINCE

In this study, we simulated two situations existed in the real world: NoTarget, the query herb with no known target (all of known targets as test targets); HalfTarget, the query herb with half of known targets (Half of known targets as train targets, the left targets as test targets). For the two settings: NoTarget and HalfTarget, we selected 261 herbs and 896 herbs as test herbs, respectively. Using LOOCV, the prediction performance of heNetRW was compared with PRINCE. Target prediction results (Table 2) implied that the performance of heNetRW was slightly better than PRINCE (improved F1-score by 0.08 and Hit@1 by 21.34%) under the NoTarget simulation. We also displayed F1-scores distribution of 261 test herbs with different number of test targets (known targets) (Fig. 4a). The distribution of F1-scores indicated that heNetRW (64.75% F1-scores bigger than 0.20) had better performance (*p*-value = 1.10e-8) than PRINCE (40% F1-scores bigger than 0.20). Under the HalfTarget simulation, heNetRW had much better performance than PRINCE (improved F1-score by 0.54 and Hit@1 by 69.08%). And the F1-scores distribution also implied heNetRW (70.87% F1-scores bigger than 0.40) has much better performance (*p*-value = 1.10e-249) than PRINCE (1.23% F1-scores bigger than 0.40). For the PRINCE, iteration algorithm would spread information of known nodes to other nodes in the PPI network. If the known nodes were isolated nodes in the PPI network, the information of the known nodes would not spread to other nodes, which would lead to poor prediction performance. But for the heNetRW algorithm, the information of known nodes still could spread to other nodes if there are neighbors (herb nodes or protein nodes) of these nodes in the heterogeneous herb-target network. The prediction results of PRINCE showed 67.30% F1-scores (12.17% for the heNetRW) was zero (Fig. 4b), which also implied heNetRW had a greater robustness than PRINCE. Otherwise, the prediction results also implied that under both the NoTarget and the HalfTarget, the query herbs with more test targets can lead to better performance of PRINCE. But for the heNetRW, more test targets of the query herb had a better performance under the NoTarget, and moderate quantity of test targets may have a better performance under the HalfTarget.

**Table 2:** The performance comparison of different prediction algorithms

Prediction simulation	Algorithm	Number of herbs	F1-score	Hit@1 (%)
NoTarget	PRINCE	260	0.16±0.17	29.23
	heNetRW	261	0.28±0.20	50.57
HalfTarget	PRINCE	896	0.05±0.10	6.70
	heNetRW	896	0.59±0.33	75.78

NoTarget and HalfTarget are two experimental simulations. PRINCE and heNetRW are prediction algorithms. Hit@1 represents the top one hit

### Parameters tuning of heNetRW

**Fig. 5:**
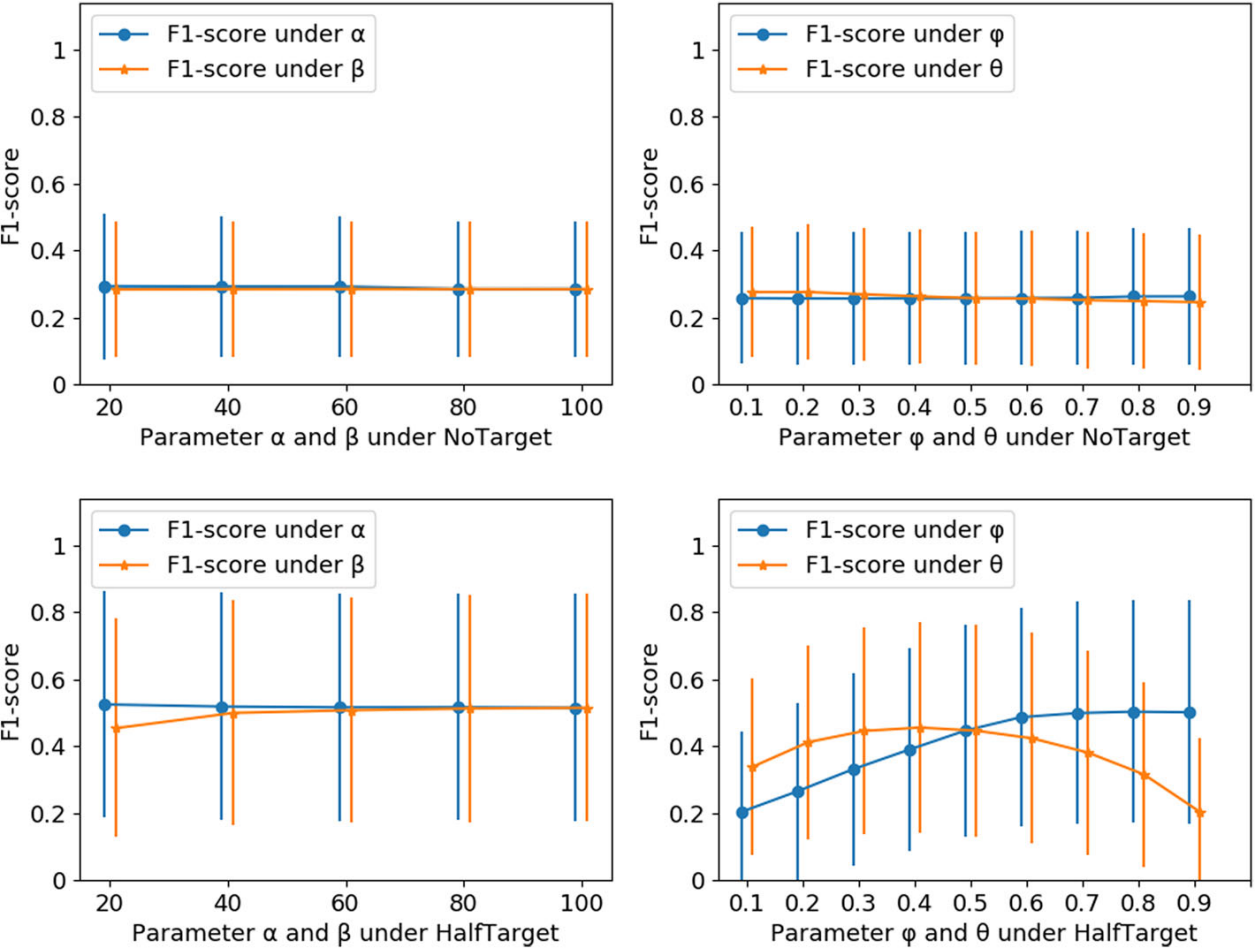
Parameter tuning of heNetRW. Under NoTarget and HalfTarget simulations, the four heNetRW-related parameters *α*, *β*, *θ* and *φ* were tuned, and F1-scores (average and standard deviation) of prediction results were showed, respectively, as follows: Fig. 5**a** tuning parameters *α* and *β* under the NoTarget; Fig. 5**b** tuning parameters *θ* and *φ* under the HalfTarget; Fig. 5**c** tuning parameters *α* and *β* under the HalfTarget; Fig. 5**d** tuning parameters *θ* and *φ* under the HalfTarget

The prediction algorithm heNetRW had four parameters need to be tuned: the number *α* of selected neighbors for each herb in the herb-herb network, the number *β* of selected neighbors for each protein in the protein-protein network, the probability *θ* to start a new journey of random walk, and the probability *φ* to jump from the herb layer to the protein layer or vice versa. For NoTarget and HalfTarget simulations, the parameter *α* and *β* were tuned at the range from 20 to 100 (scale is 20), and the parameter *θ* and *φ* were tuned at the range from 0.1 to 0.9 (scale is 0.1) (Fig. 5). When one parameter was tuned, other three parameters remain unchanged. The prediction results indicated the algorithm is not sensitive to the parameters *α*, *β*, *θ* and *φ* under the NoTarget simulation (Fig. 5a and b). And under the HalfTarget simulation, the parameters *α* and *β* have little influence on improving prediction performance, small *α* and large *β* could make heNetRW have a better prediction performance (Fig. 5c). The parameters *θ* and *φ* have much influence on prediction performance of heNetRW (Fig. 5d). The larger parameter *φ* can lead to the better prediction performance. When the parameter *θ* is 0.4, heNetRW had better prediction performance.

### Case study

**Fig. 6:**
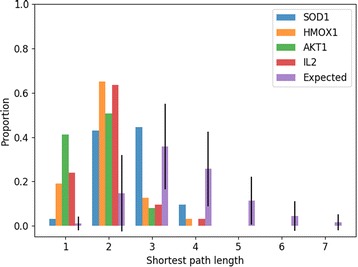
Shortest path analysis of candidate targets of rhizoma coptidis. The horizontal axis represents shortest path length, and the vertical axis represents proportion of SPLs

We took prediction results of rhizoma coptidis and turmeric as an example (Table 3). For the NoTarget simulation, all of known targets of the given herb would be regarded as test targets. And half of known targets would be regarded as test targets for the HalfTarget simulation. Under the NoTarget, there were 35 known targets of rhizoma coptidis in the top 64 candidate targets (precision/recall/F1-score = 0.55) (Table 3). Also, we listed the top 20 candidate targets of rhizoma coptidis (Table 4). 16 of top 20 candidate target (precision = 0.80; recall = 0.22) were known targets (the bold targets in the Table 4) of rhizoma coptidis. To fully evaluate the performance of heNetRW, we consulted recent published papers to verify whether recent researches indicated the left 4 targets were associated to rhizoma coptidis. The researches of Han et al. [28] and Huang et al. [29] implied CYP3A4 (rank = 7) and ICAM1 (rank = 18) were related to rhizoma coptidis, respectively. For the turmeric with more test targets (152 known targets as test targets), 81 known targets appeared in the top 152 candidate targets (precision/recall/F1-score = 0.53). The entire top 20 predicted candidate targets (Table 4) were known targets of turmeric (precision = 1; recall = 0.13). As for the HalfTarget simulation, there were 24 and 43 known targets of rhizoma coptidis (precision/recall/F1-score = 0.75) and turmeric (precision/recall/F1-score = 0.57) in the top 32 and 76 candidate targets, respectively (Table 3). There were 17 known targets and 18 known targets of rhizoma coptidis (precision = 0.85; recall = 0.53) and turmeric (precision = 0.90; recall = 0.24) in the top 20 candidate targets, repectively (Table 5). Li et al. [30] revealed that coptisine reduced the expression of the MMP9 (rank = 16) at the mRNA level. By RT-qPCR and Western blot data, Wang et al. [31] showed that curcumol phosphorylated CDK2 (rank = 12) and CDK4 (rank = 14), which indicated that both of candidate targets are related to turmeric. For the left four candidate targets: SOD1 (NoTarget, rank = 8), HMOX1 (NoTarget, rank = 19), AKT1 (HalfTarget, rank = 12), and IL2 (HalfTarget, rank = 17), the average shortest path length (ASPL) and the distribution of shortest path length (SPL) analysis between these candidate targets and known targets of rhizoma coptidis (named KTofRC) were conducted (Fig. 6). Compared with random experiment (ASPL = 3.74±0.86), the ASPL between these candidate targets (SOD1: 2.60; HMOX1: 2.00; AKT1: 1.67; IL2: 1.92) and KTofRC indicated that these targets had strong interaction with KTofRC in the PPI network. The SPLs between these candidate targets and KTofRC were mainly distributed 1 and 2, e. g. for SOD1, HMOX1, AKT1 and IL2, 46.03%, 84.13%, 92.06% and 87.30% of SPLs with smaller than 3 (15.55% of SPLs for the random shuffle), which also implied that there were strong interaction between these targets and KTofRC.

**Table 3:** The prediction performance of rhizoma coptidis and turmeric

Simulation	Herb	Number of Train targets	Number of test targets	Number of correct targets	Precision/recall /F1-score
NoTarget	Rhizoma coptidis	0	64	35	0.5469
	Turmeric	0	152	81	0.5329
HalfTarget	Rhizoma coptidis	32	32	24	0.75
	Turmeric	76	76	43	0.5658

PRINCE and heNetRW are algorithms of herb target prediction

**Table 4:** The top 20 candidate targets of rhizome coptidis and turmeric under the NoTarget

Rhizoma coptidis			Turmeric		
Rank	Candidate Target	Score	Rank	Candidate Target	Score
1	**CASP3**	0.4526	1	**CASP3**	0.2750
2	**RELA**	0.4109	2	**RELA**	0.2554
3	**PTGS2**	0.3834	3	**PTGS2**	0.2222
4	**TNF**	0.2988	4	**TNF**	0.1816
5	**NOS2**	0.2771	5	**BCL2**	0.1716
6	**BCL2**	0.2708	6	**NOS2**	0.1624
7	CYP3A4	0.2325	7	**BAX**	0.1403
8	SOD1	0.2132	8	**TP53**	0.1349
9	**BAX**	0.2096	9	**JUN**	0.1318
10	**CASP9**	0.1928	10	**CYP3A4**	0.1230
11	**IL6**	0.1846	11	**SOD1**	0.1182
12	**JUN**	0.1777	12	**FOS**	0.1165
13	**CDKN1A**	0.1701	13	**CASP9**	0.1158
14	**TP53**	0.1701	14	**VEGFA**	0.1141
15	**IL1B**	0.1658	15	**IL6**	0.1111
16	**VEGFA**	0.1612	16	**CDKN1A**	0.1106
17	**FOS**	0.1561	17	**NFKBIA**	0.0992
18	ICAM1	0.1558	18	**IL1B**	0.0926
19	HMOX1	0.1502	19	**MMP9**	0.0873
20	**MAPK1**	0.1358	20	**ICAM1**	0.0872

The bold candidate targets are known targets of given herbs

**Table 5:** The top 20 candidate targets of rhizome coptidis and turmeric under the HalfTarget

Rhizoma coptidis			Turmeric		
Rank	Candidate Target	Score	Rank	Candidate Target	Score
1	**PTGS2**	0.0311	1	**CASP3**	0.0657
2	**NOS2**	0.0285	2	**TNF**	0.0619
3	**BAX**	0.0259	3	**BCL2**	0.0518
4	**CASP9**	0.0252	4	**JUN**	0.0438
5	**CDKN1A**	0.0238	5	**VEGFA**	0.0424
6	**NFKBIA**	0.0220	6	**BAX**	0.0400
7	**FOS**	0.0212	7	**IL6**	0.0390
8	**CDK2**	0.0203	8	**MMP9**	0.0355
9	**VEGFA**	0.0202	9	**CASP9**	0.0334
10	**IL4**	0.0178	10	**AKT1**	0.0326
11	**BCL2L1**	0.0160	11	**CCND1**	0.0302
12	AKT1	0.0146	12	CDK2	0.0296
13	**HERC5**	0.0143	13	**MAPK1**	0.0282
14	**CDC2**	0.0135	14	CDK4	0.0269
15	**EIF6**	0.0132	15	**XDH**	0.0245
16	MMP9	0.0130	16	**SOD1**	0.0241
17	IL2	0.0126	17	**PRKCB**	0.0233
18	**MPO**	0.0121	18	**CYP3A4**	0.0213
19	**EGFR**	0.0120	19	**VCAM1**	0.0211
20	**HIF1A**	0.0118	20	**MYC**	0.0202

The bold candidate targets are known targets of given herbs

## Discussion

In genetic research, as a universal amplifier of genetic associations [32], network propagation methods, e.g. random walk [25], information diffusion [33] and electrical resistance [34], have been applied successfully to identify gene function [35], disease characterization [36], and drug targets [37]. In our study, a random walk algorithm on the heterogeneous herb-target network has been proposed to identify candidate targets for herbs. By building a heterogeneous herb-target network and simulating random walk on the network, the candidate targets of given herbs can be predicted. In the experiment stage, the NoTarget and HalfTarget situations were simulated to validate prediction performance of heNetRW. The final results indicated that heNetRW had better prediction performance than PRINCE.

There are two advantages of heNetRW algorithm. The algorithm PRINCE simulated random walk on the PPI network, and the initial nodes are known proteins of the herbs that are relevant to query herb. Nevertheless, for the heNetRW, random walk was simulated on the heterogeneous herb-target network, the initial nodes are targets that are relevant to known targets of the query herb and herbs that are relevant to the query herb. So with respect to PRINCE considering herb similarities and PPI associations, heNetRW considered more known information including known herb-target associations and pathway-based protein similarities. Experimental results also indicated more known information could make heNetRW have better prediction performance. Otherwise, by tuning parameters of heNetRW algorithm, we effectively controlled the trend of random walk to improve prediction performance. The prediction results can be used to guide the selection of candidate targets of herbs that have not been studied at present or find new protein targets of common herbs, which would help to reveal molecular mechanism of herbal drugs and improve treatment of complex diseases.

In recent years, network embedding representation methods, e.g. deepwalk [38], LINE [39] and node2vec [40], have been widely applied to network classification [41] and link prediction [40]. By learning continue feature representations for nodes in networks, the node2vec algorithm can obtain a mapping of nodes to a low-dimensional space of features [40], which can be used to measure accurate similarity of nodes. In our study, the similarities of herb pairs and target pairs are based on efficacy-based and pathway-based cosine similarities, which only considered unilateral similarity of herb pairs or target pairs. In the future, by building heterogeneous herb-related (e.g. efficacies, indications and ingredients) network and target-related (e.g. pathways, GO terms and interactions) network, we will apply network embedding algorithm to obtain multi-dimensional similarity measure of herb pairs or target pairs. Otherwise, Under the NoTarget simulation, since initial nodes only contain herb nodes, not target nodes, the prediction performance of heNetRW was not well. To address the issue, known targets of herbs that are relevant to the query herb will be added to the set of initial nodes in the future.

## Conclusions

Herb target identification is a critical step for revealing pharmacological mechanisms of herbal drugs and improving clinical treatment of diseases in TCM. In this study, we developed a heterogeneous network propagation method to identify herb targets. Based on two validation settings, our method was compared with the baseline method PRINCE. The experimental results indicated that our method had higher performance than PRINCE. We manually evaluated several candidate targets of two herbs, which is not in benchmark dataset, but have been confirmed by recent published papers. Therefore, the prediction results not only can be used to guide the selection of herbal candidate targets in wet lab, but also help to reveal molecule mechanisms of herbs.

## Additional files

Additional file 1: **–** HIT_herb_target.xls. 23,453 herb-target associations between 1016 herbs and 1214 targets were collected and integrated from the HIT database. (XLS 1200 kb)

Additional file 2: **–** CHPA_herb_efficacy.xls. 3487 herb-efficacy associations between 742 herbs and 360 efficacies were collected from the Chinese pharmacopoeia (CHPA, 2015 edition). (XLS 200 kb)

Additional file 3: **–** KEGG_protein_pathway.xls. 16,162 protein-pathway associations between 4794 proteins and 244 pathways were collected from KEGG database. (XLS 896 kb)

## Abbreviations

ASPL: average shortest path length
CHPA: Chinese pharmacopoeia
GO: gene ontology
HeNetRW: random walk method on heterogeneous herb-target network for herb target prediction
Hit@1: the top one hit
KTofRC: known targets of rhizome coptidis
LOOCV: leave one out cross validation
NoTarget and HalfTarget: validation settings for prediction algorithms
PPI: protein-protein interaction
SPL: shortest path length
